# Severity in *Plasmodium vivax* malaria claiming global vigilance and exploration – a tertiary care centre-based cohort study

**DOI:** 10.1186/1475-2875-13-304

**Published:** 2014-08-08

**Authors:** Kavitha Saravu, Kumar Rishikesh, Asha Kamath, Ananthakrishna B Shastry

**Affiliations:** Department of Medicine, Kasturba Medical College, Manipal University, Manipal, 576104 Karnataka India; Department of Community Medicine, Kasturba Medical College, Manipal University, Manipal, India

**Keywords:** *Plasmodium vivax*, *Plasmodium falciparum*, Malaria, Vivax malaria, Falciparum malaria, Parasitaemia, Severity, Cerebral malaria, Anti-malarials

## Abstract

**Background:**

Mounting reports on severe *Plasmodium vivax* malaria from across the globe have raised concerns among the scientific community. However, the risk of *P. vivax* resulting in complicated malaria and mortality is not as firmly established as it is with *Plasmodium falciparum*. This study was conducted to determine the severity proportion and factors associated with severity in cases of vivax and falciparum malaria.

**Methods:**

Adult patients microscopically diagnosed to have *P. vivax/P. falciparum* infections from the year 2007-2011 were evaluated based on their hospital records. Severe malaria was defined as per the World Health Organization’s guidelines. Comparison was made across species and binary logistic regression was used to determine risk factors of severity.

**Results:**

Of 922 malaria cases included in the study, *P. vivax* was the largest (63.4%, 95% confidence interval (CI) 60.3-66.5%) infecting species, followed by *P. falciparum* (34.4%, 95% CI 31.3-37.5%) and their mixed infection (2.2%, 95% CI 1.3-3.2%). Severity in *P. vivax* and *P. falciparum* was noted to be 16.9% (95% CI 13.9-19.9%) and 36.3% (95% CI 31.0-41.6%) respectively. *Plasmodium falciparum* had significantly higher odds [adjusted odds ratio (95% CI), 2.80 (2.04-3.83)] of severe malaria than *P. vivax*. Rising respiratory rate [1.29 (1.15-1.46)], falling systolic blood pressure [0.96 (0.93-0.99)], leucocytosis [12.87 (1.43-115.93)] and haematuria [59.36 (13.51-260.81)] were the independent predictors of severity in *P. vivax.* Increasing parasite index [2.97 (1.11-7.98)] alone was the independent predictor of severity in *P. falciparum*. Mortality in vivax and falciparum malaria was 0.34% (95% CI -0.13-0.81%) and 2.21% (95% CI 0.59-3.83%), respectively. Except hyperparasitaemia and shock, other complications were associated (P < 0.05) with mortality in falciparum malaria. Pulmonary oedema/acute respiratory distress syndrome was associated (P = 0.003) with mortality in vivax malaria. Retrospective design of this study possesses inherent limitations.

**Conclusions:**

*Plasmodium vivax* does cause severe malaria and mortality in substantial proportion but results in much lesser amalgamations of multi-organ involvements than *P. falciparum*. Pulmonary oedema/acute respiratory distress syndrome in *P. vivax* infection could lead to mortality and therefore should be diagnosed and treated promptly. Mounting complications and its broadening spectrum in ‘not so benign’ *P. vivax* warrants global vigilance for any probable impositions.

**Electronic supplementary material:**

The online version of this article (doi:10.1186/1475-2875-13-304) contains supplementary material, which is available to authorized users.

## Background

The component of ‘severity’ or ‘complication’ in malaria governs the choice of anti-malarial regimen and warrants intensive management. *Plasmodium falciparum* is entrenched to be associated with life-threatening complications, leading to widespread global annual mortality. There has been a gradual increase in reports describing sporadic atypical manifestations [1–4] to wide spectrum severity [5–7] among *Plasmodium vivax* patients from India and other parts of the globe. Of late this clamour on the rising severity in *P. vivax* malaria has raised concerns among the scientific community. However, the risk of *P. vivax* resulting in complicated malaria and mortality is not firmly established [8]. The spectrum of malarial severity may be extensively different among various populations with diverse endemicity [9]. In India, there have been few investigations to determine the spectrum of severity and its associated factors with *P. vivax* in comparison to *P. falciparum*. In the existing literature from India and elsewhere, studies have been either case reports [1–3], or have lacked a denominator or comparison with other malaria species [7] or multivariate logistic regression analysis [6, 7, 10], or have described exiguous complications [11].

This study was aimed to perform a robust statistical analysis to determine the spectrum of severity, its relative proportions and factors associated with severity among vivax and falciparum malaria and assess the validity of the ‘benign’ tag with *P. vivax*.

## Methods

### Study design and patients

**Figure 1:**
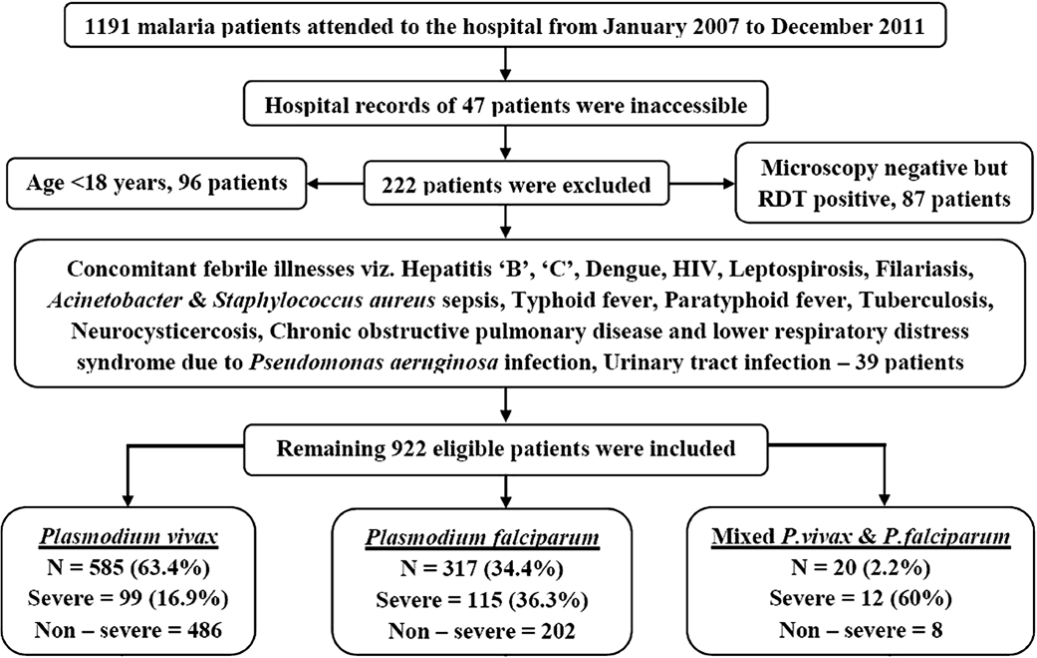
**Flow chart of patients’ selection and distribution of severe malaria across species.** RDT = Rapid diagnostic test.

A study based on the records in a tertiary care hospital was conducted from January 2007 to December 2011. Initially patients’ data were abstracted onto physical proforma and verified manually followed by constructing an electronic database, its validation and analysis. Patients of either gender, ≥18 years, hospitalized with acute malaria, diagnosed by both quantitative buffy coat and Leishman’s stained peripheral blood smears with the presence of asexual forms of *P. vivax*, *P. falciparum* or both, with or without gametocytes, were included. All cases with, co-existent, non-malarial febrile illnesses were excluded, Figure 1. Patients were managed by hospital physicians as per their clinical judgement and national guidelines.

### Ethics statement

Prior to the commencement of the study, an approval of the ethics committee of the Kasturba hospital, Manipal University, Manipal, Karnataka, India was obtained. In view of retrospective design of the study, obtaining individual patients’ consent was not deemed necessary by the ethics committee; however, all patients’ records were kept anonymized.

### Variables

#### Independent variables

Disease severity or complications were defined as per the guidelines laid by the World Health Organization (WHO) for the management of severe malaria in the year 2012 [12], with a modification for clinical jaundice/liver dysfunction, i e, rise in total bilirubin ≥2.5 mg/dL with simultaneous three-fold elevation in any serum aminotransferases from their reference upper limits. Severity determinants were restricted to cerebral malaria (impaired consciousness, coma or multiple generalized convulsions within 24 hours), liver dysfunction, pulmonary oedema (PE) or acute respiratory distress syndrome (ARDS) (radiological), renal failure (serum creatinine >3 mg/dL), shock (systolic blood pressure <80 mm Hg), spontaneous bleeding, hyperparasitaemia (parasite index >5%, i e, percentage of parasitized erythrocytes on Leishman-stained peripheral blood smear), hypoglycaemia (blood sugar <40 mg/dL), respiratory distress (respiratory rate >32 beats/minute), metabolic acidosis (plasma bicarbonate <15 mmol/L) and severe anaemia (haemoglobin <7 g/dL).

#### Dependent variable

Diagnosis of severe/complicated malaria was the primary outcome. The entire cohort was classified into severe/complicated and non-severe/uncomplicated malaria stratified by the infecting species. Supportive requirements including intensive care, more than seven days of hospitalization and in-hospital mortality were secondary outcomes.

### Statistical analysis

Categorical data were summarized as frequency and proportions by severity category for each malaria species. Proportions were examined using *χ*^2^ test or Fisher’s exact test. Continuous variables were tested for normality using the Kolmogorov–Smirnov test. Normally distributed continuous variables were reported as means with standard deviations (SD), and compared by independent sample *t*-test. Skewed variables were summarized as medians with interquartile range (IQR) and compared by Mann Whitney *U* test. Logistic regression analyses were performed: 1) to determine the factors associated with disease severity; and, 2) to determine the odds of supportive requirements and mortality by disease severity and malaria species. Multicollinearity was tested with variables, which yielded a P-value of ≤0.1 in univariate analysis. Out of any pair of variable showing collinearity, only one variable was included into the multivariate logistic regression model. All tests of significance were two-sided, with a P-value of <0.05 indicating statistical significance. Data analysis was done using Statistical Package for the Social Sciences version 15.0 (SPSS, South Asia, Bangalore, India).

## Results

There were total 3,00,150 in-hospital admissions from January 2007 to December 2011 including 1,79,842 males and 1,20,308 females. A total of 1,191malaria patients (0.40%) were hospitalized during the same period. Hospital records of 47 (3.1%) patients remained inaccessible and 222 (19.40%) cases were excluded in accordance with pre-specified criteria. Remaining 922 eligible cases were included for the study. *Plasmodium vivax* was the largest (63.4%, 585/922) infecting malaria species followed by *P. falciparum* (34.4%, 317/922) and their mixed infections (2.2%, 20/922) (Figure 1). Due to a relatively smaller cohort of mixed infection, they could not be included in any further analysis.

### Demographic, clinical and laboratory characteristics by malaria species

**Figure 2:**
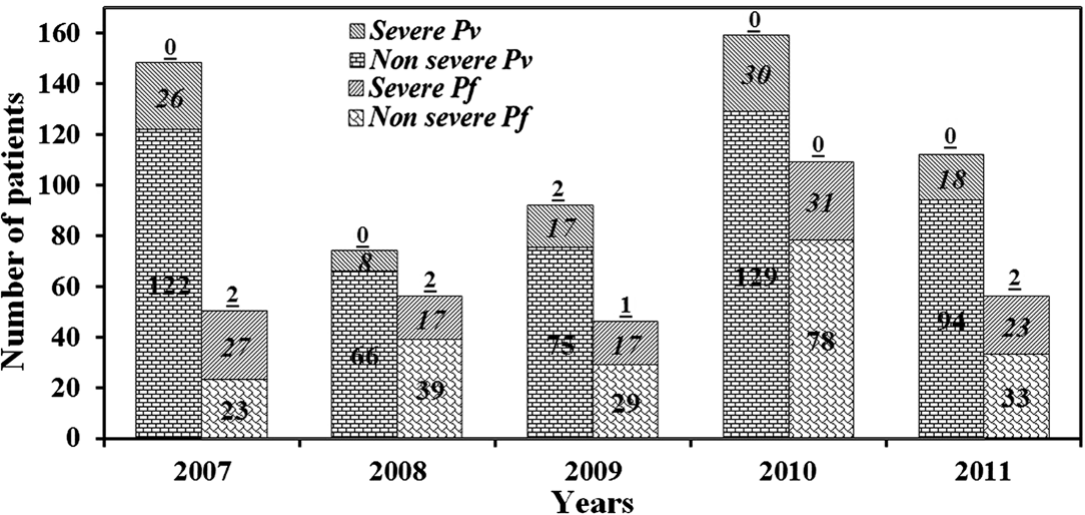
**Year-wise proportion and fluctuation in complicated** ***Plasmodium vivax*** **and** ***Plasmodium falciparum*** **cases included for the study.** Lower stack of each column represents number of total non-severe cases (bold font) for the respective year, upper stack of each column represents number of severe cases (bold and italic font) for the respective year, above each column the bold and underlined font represents number of mortality with respective species for the year.

Proportions of complicated cases among *P. vivax* and *P. falciparum* were 16.9% (99/585) and 36.3% (115/317), respectively (P <0.001). Figure 2 depicts the year-wise proportions with rise and fall in complicated *P. vivax* and *P. falciparum* cases included for the study. Male preponderance (>70%) was observed across all complicated malaria cohorts with no significant difference in interspecies gender distribution (P = 0.26). The mean duration of hospitalization in *P. vivax* and *P. falciparum* was 5.3 ± 2.4 days and 7.4 ± 3.6 days, respectively (P <0.001).

### Plasmodium vivax

Patients with complicated *P. vivax* were significantly older (median age in years 40 vs. 26.5), P <0.001 (Additional file 1). Duration of fever (P <0.001), frequency of pallor (P = 0.02), icterus (P <0.01) and hepatomegaly (P = 0.02) were significantly greater in complicated *P. vivax* cohort. Systolic blood pressure (P = 0.04), axillary temperature (P = 0.002), haemoglobin and platelet count (P <0.001) were significantly lower in complicated *P. vivax*. There were significant (P <0.001) elevations in leucocyte count, erythrocyte sedimentation rate, levels of total and direct bilirubin, serum aspartate aminotransferase, serum alkaline phosphatase, blood urea and frequency of haematuria, and haemoglobinuria in complicated *P. vivax*. Occurrence of leucocytosis was also found to be associated with complicated *P.vivax* malaria (P = 0.003).

### Plasmodium falciparum

History of fever and headache was significantly less frequent (P = 0.03) in complicated *P. falciparum* cohort. Diarrhoea (P = 0.002), pallor and icterus (P <0.001), splenomegaly (P = 0.03) and hepatomegaly (P = 0.01) were more frequent in complicated *P. falciparum*. Respiratory rate (P <0.001) and parasite index (P = 0.014) were found to be significantly elevated in complicated *P. falciparum* cohort. Haemoglobin (P <0.001) and platelet count (P = 0.002) were significantly decreased in complicated *P. falciparum*. Leucocyte count, haematuria, haemoglobinuria, erythrocyte sedimentation rate, total and direct bilirubin, aspartate aminotransferase, blood urea, and serum creatinine had significantly (P <0.001) increased in complicated *P. falciparum*. Occurrence of leucocytosis was also found to be associated with complicated *P. falciparum* malaria (P <0.001).

### Patients’ treatment

Patients were initiated on anti-malarials only after confirmed microscopic diagnosis of malarial infections. Majority (75%) of *P. falciparum*, complicated *P. vivax*, and mixed infections were treated with artesunate combination therapies, although 25% patients received standard seven-day parenteral quinine in combination with doxycycline/sulphadoxine-pyrimethamine till the year 2009. The year 2010 witnessed an abrupt change in anti-malarial treatment choice by clinicians, when quinine prescription waned off and artesunate based combination therapy remained the only choice for all cases of *P. falciparum*, mixed infections and complicated *P. vivax*. Uncomplicated *P. vivax* cases were treated with standard oral chloroquine and primaquine regimen.

Until preliminary febrile investigations and culture reports were obtained, empiric antibiotics were administered to 6.6% (32/486), 8.4% (17/202) and 12.5% (1/8) uncomplicated cases and to 9.1% (9/99), 17.4% (20/115) and 25% (3/12) complicated cases of *P. vivax, P. falciparum* and mixed malaria, respectively. Significantly high (P = 0.003) proportion [14.2% (32/226)] of patients with complicated malaria had received empiric antibiotics than uncomplicated malaria [7.2% (50/696)].

### Association of complications with mortality

Occurrence of cerebral malaria, liver dysfunction, severe anaemia (P = 0.002), haemoglobinuria (P = 0.04) and renal failure (P <0.001) was significantly higher in *P. falciparum* than *P. vivax*. Hyperparasitaemia was significantly (P = 0.002) associated with *P. falciparum* only. In *P. vivax* mortality was significantly associated with PE/ARDS (P = 0.003) and respiratory distress (P = 0.001). In *P. falciparum* except shock and hyperparasitaemia, mortality was significantly associated with all the other severity determinants (Table 1).

**Table 1:** **Association of complications with mortality by malaria species**

Malaria species	*Plasmodium vivax*(N = 585)			*Plasmodium falciparum*(N = 317)			P-value*
Determinants of severity	Complicated N (%) 99 (16.90%)	Mortality N (%) 2 (0.34%)	P - value ^#^	Complicated N (%) 115 (36.30%)	Mortality N (%) 7 (2.21%)	P-value ^#^	<0.001
Cerebral malaria	4 (4)	0	1.00	20 (17.4)	4 (57.1)	**<0.001**	**0.002**
Spontaneous bleeding	35 (35.4)	0	1.00	31 (27.0)	4 (57.1)	**0.002**	0.24
PE/ARDS	34 (34.3)	2 (100)	**0.003**	29 (25.2)	4 (57.1)	**0.002**	0.18
Shock	3 (3)	0	1.00	7 (6.1)	0	1.00	0.35
Liver dysfunction	11 (11.1)	0	1.00	33 (28.7)	4 (57.1)	**0.003**	**0.002**
Severe anaemia	6 (6.1)	0	1.00	25 (21.7)	4 (57.1)	**0.001**	**0.002**
Haemoglobinuria	6 (6.1)	0	1.00	17 (14.9)	2 (28.6)	**0.049**	**0.04**
Renal failure	6 (6.1)	0	1.00	38 (33)	5 (71.4)	**<0.001**	**<0.001**
Respiratory distress	18 (18.2)	2 (100)	**0.001**	16 (13.9)	2 (28.6)	**0.04**	0.46
Metabolic acidosis	3 (3)	0	1.00	8 (7)	3 (42.9)	**<0.001**	0.23
Hyperparasitaemia	0	0	‡	10 (8.7)	1 (14.3)	0.20	**0.002**
Hypoglycaemia	0	0	‡	2 (1.7)	2 (28.6)	**0.001**	0.50

*P - value obtained for difference in complications across malaria species by either chi - square or Fischer’s exact test, significant values, i e, <0.05 are shown in bold face.

^#^P - value obtained for association of mortality with each complications by malaria species by either Chi-square or Fischer’s exact test, significant values, i e, <0.05 are shown in bold face.

^‡^Statistics could not be performed.

Both *P. vivax* patients succumbed due to ARDS and respiratory distress. Out of seven *P. falciparum* patients who died, only one succumbed with single complication, i e, acute kidney injury despite dialysis. More than one complication resulted in mortality in other fatalities with *P. falciparum*. In both mixed malaria patients who died, more than one complication was associated with mortality (Additional file 2). None of the patients who died had any co-morbid aetiology.

### Association of demographic, clinical and laboratory factors with ‘complicated malaria’ as an outcome by malaria species

With reference to *P. vivax,* the likelihood of developing complicated malaria was significantly higher in *P. falciparum* [odds ratio - 2.80 confidence interval - (2.04-3.83)]. In *P. vivax*, increasing respiratory rate [1.29 (1.15-1.46)], falling systolic blood pressure [0.96 (0.93-0.99)], leucocytosis [12.87 (1.43-115.93)] and haematuria [59.36 (13.51-260.81)] were the independent predictors of complicated malaria. In *P. falciparum*, rising parasite index [2.97 (1.11-7.98)] was found to be the only independent predictor of complicated malaria (Table 2).

**Table 2:** **Logistic regression analysis with clinico-demographic and laboratory characteristics for determining the associated factor/s with complicated malaria**

Malaria species	*Plasmodium vivax*(N = 585)				*Plasmodium falciparum*(N = 317)			
Characteristics	Odds ratio (95% CI)	P-value*	Adjusted Odds ratio (95% CI)	P-value*	Odds ratio (95% CI)	P-value*	Adjusted Odds ratio (95% CI)	P-value*
**Malaria species** ^**ŧ**^	**Reference**				**2.80 (2.04-3.83)**	**<0.001**		
***Demographic***								
Gender, male	**0.49 (0.29-0.81)**	**0.005**	0.37 (0.09-1.53)	0.17	0.87 (0.49-1.55)	0.63	§	
Age, years (median, IQR)	**1.03 (1.02-1.05)**	**<0.001**	#		1.01 (0.99 - 1.03)	0.11	§	
Age categories								
Up to 40 years	Reference		Reference		Reference			
Between 41 - 60 years	**2.12 (1.31-3.42)**	**0.002**	1.56 (0.49-4.97)	0.45	1.12 (0.69-1.84)	0..65	§	
More than 60 years	**2.40 (1.06-5.44)**	**0.035**	2.59 (0.41-16.16)	0.31	1.64 (0.57-4.70)	0.36	§	
History of fever	0.82 (0.09-7.37)	0.86	§		**0.11 (0.01-0.94)**	**0.04**	‡	
More than 3 days of fever	**2.25 (1.35-3.77)**	**0.002**	2.30 (0.62-8.55)	0.21	1.58 (0.88-2.85)	0.13	§	
Headache	**0.56 (0.36-0.87)**	**0.01**	1.24 (0.44 - 3.49)	0.69	**0.58 (0.36-0.92)**	**0.02**	‡	
Diarrhoea	1.43 (0.56-3.63)	0.46	§		**4.83 (1.94-12.04)**	**0.001**	‡	
Pallor	**1.97 (1.13-3.44)**	**0.02**	#		**4.35 (2.60-7.29)**	**<0.001**	#	
Icterus	**2.48 (1.51-4.08)**	**<0.001**	#		**3.53 (2.18-5.71)**	**<0.001**	#	
Splenomegaly	0.83 (0.52-1.32)	0.44	§		**1.63 (1.02-2.61)**	**0.04**	‡	
Hepatomegaly	**1.80 (1.12-2.89)**	**0.015**	0.55 (0.16-1.91)	0.35	**1.86 (1.16-2.97)**	**0.01**	0.03 (0.00-9.20)	0.23
Pulse rate (/min)	**1.02 (1.001-1.03)**	**0.036**	1.01 (0.97-1.04)	0.81	1.01 (0.99-1.02)	0.40	§	
Respiratory rate (/min)	**1.11 (1.06-1.17)**	**<0.001**	**1.29 (1.15-1.46)**	**<0.001**	**1.09 (1.04-1.13)**	**<0.001**	0.98 (0.66-1.46)	0.920
Systolic blood pressure (mmHg)	**0.98 (0.96-0.99)**	**0.012**	**0.96 (0.93-0.99)**	**0.02**	0.99 (0.98-1.01)	0.74	§	
Diastolic blood pressure (mmHg)	**0.98 (0.96-0.99)**	**0.02**	#		0.99 (0.97-1.01)	0.34	§	
Axillary temperature (°F)^a^	**0.85 (0.74-0.97)**	**0.014**	0.78 (0.56-1.09)	0.150	0.86 (0.74-1.01)	0.06	§	
Parasite index (%)	1.66 (0.74-3.69)	0.22	§		**1.33 (1.07-1.66)**	**0.01**	**2.97 (1.11-7.98)**	**0.03**
Hemoglobin (gm/dL)	**0.77 (0.70-0.85)**	**<0.001**	0.86 (0.67-1.11)	0.24	**0.76 (0.69-0.84)**	**<0.001**	0.87 (0.39-1.94)	0.73
Leucocyte count/1,000/mm^3^	**1.23 (1.12-1.34)**	**<0.001**	#		**1.08 (1.01-1.15)**	**0.03**	#	
Leucocytosis ≥11,000/mm^3^	**4.78 (1.79-12.74)**	**0.002**	**12.87 (1.43-115.93)**	**0.020**	**9.17 (3.63-23.17)**	**<0.001**	‡	
Leucopenia ≤4,000/mm^3^	0.72 (0.40-1.31)	0.28	§		**0.36 (0.17-0.75)**	**0.006**	0.48 (0.01-17.62)	0.690
Platelet count/10,000/mm^3^	**0.95 (0.91-0.99)**	**0.012**	#		0.97 (0.93-1.002)	0.06	§, #	
Thrombocytopaenia ≤40,000/mm^3^	**3.28 (1.99-5.41)**	**<0.001**	2.44 (0.67-8.88)	0.180	**2.14 (1.30-3.52)**	**0.003**	3.96 (0.17-91.31)	0.39
Erythrocyte sedimentation rate/10 (/1^st^ hour)	**1.16 (1.08-1.24)**	**<0.001**	#		**1.19 (1.11-1.27)**	**<0.001**	#	
Total bilirubin (mg/dL)	**1.18 (1.09-1.28)**	**<0.001**	1.11 (0.93-1.32)	0.27	**1.14 (1.09-1.19)**	**<0.001**	1.36 (0.93-1.98)	0.11
Direct bilirubin (mg/dL)	**1.22 (1.11-1.35)**	**<0.001**	#		**1.18 (1.11-1.25)**	**<0.001**	#	
Aspartate aminotransferase (IU/L)	**1.01 (1.01-1.02)**	**<0.001**	1.01 (0.99-1.02)	0.28	**1.02 (1.01-1.02)**	**<0.001**	1.02 (0.98-1.06)	0.330
Alanine aminotransferase (IU/L)	1.01 (1.00-1.01)	0.06	§, #		**1.01 (1.001-1.011)**	**0.02**	#	
Alkaline phosphatase (U/L)	**1.01 (1.01-1.02)**	**<0.001**	1.00 (0.99-1.01)	0.21	**1.01 (1.002-1.01)**	**0.002**	0.98 (0.94-1.03)	0.43
Blood urea (mg/dL)	**1.03 (1.02-1.05)**	**<0.001**	#		**1.02 (1.01-1.03)**	**<0.001**	#	
Serum creatinine (mg/dL)	**2.78 (1.64-4.72)**	**<0.001**	2.20 (0.90-5.40)	0.09	**3.51 (2.26-5.44)**	**<0.001**	#	
Haematuria ≥ 0-5 RBCs/hpf	**23.74 (9.74-57.83)**	**<0.001**	**59.36 (13.51-260.81)**	**<0.001**	**7.41 (2.99-18.33)**	**<0.001**	‡	

^ŧ^Univariate analysis considering malaria species as independent and complicated malaria as dependent variable.

^§^Univariate model did not yield significant association.

^‡^Statistics could not be computed.

*Significant P-value, i e, <0.05 are shown in bold face and odds ratios which did not overlap the null value, i e, 1 are shown in bold face.

^a^To convert temperature to ^0^C=[0F-32]*5/9.

^#^Indicates collinearity, In *P. vivax*, Age - Age Category, Pallor - Haemoglobin and Erythrocyte sedimentation rate/10, Icterus - Total bilirubin, Systolic blood pressure - Diastolic blood pressure, Haemoglobin - Erythrocyte sedimentation rate/10, Leucocyte count/1,000 - Leucocytosis, Platelet count/10,000 - Thrombocytopaenia, Total bilirubin - Direct bilirubin, Blood urea - Direct bilirubin and Serum creatinine, Aspartate aminotransferase - Alanine aminotransferase. In *P. falciparum*, Pallor - Haemoglobin and Erythrocyte sedimentation rate/10, Icterus - Total and Direct bilirubin, Hemoglobin - Erythrocyte sedimentation rate/10, Leucocyte count/1000 - Leucocytosis, Platelet count/10000 - Thrombocytopaenia, Total bilirubin - Direct bilirubin, Total and Direct bilirubin - Blood urea and Serum creatinine, Alanine aminotransferase - Aspartate aminotransferase, Blood urea - Leucocytosis and Serum creatinine.

### Association of malaria severity as predictor for supportive requirements and outcomes by malaria species

**Figure 3:**
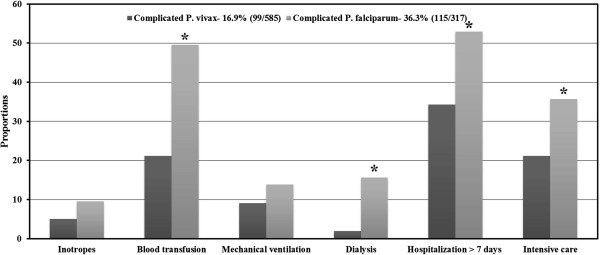
**Association of supportive requirements by complicated malaria species.** * = P <0.05.

Compared to uncomplicated cohort, odds of blood transfusion [9.80 (4.71-20.37)] and [10.70 (5.77-19.82)], more than seven days of hospitalization [6.54 (3.83-11.18)] and [2.72 (1.70-4.38)], and prolonged duration of intensive care [4.19 (1.79-9.78)] and [1.91(1.47-2.48)] was more in both complicated *P. vivax* and *P. falciparum,* respectively. Among blood products transfused, odds of platelet [10.61 (4.31-26.13)] and [7.44 (3.60-15.60)] and packed red cells [6.06 (1.49-24.75)] and [13.40 (4.84-37.11)] were significantly higher in complicated *P. vivax* and *P. falciparum,* respectively. Figure 3 depicts the proportions of supportive requirements in *P. vivax* and *P. falciparum* cohorts. Mortality occurred in 2% (2/99), 6.1% (7/115) and 16.7% (2/12) of severe *P. vivax*, *P. falciparum* and mixed infection, respectively. There was no difference in mortality either across complicated groups of all species (P = 0.06) or between *P. vivax* and *P. falciparum* (P = 0.18).

## Discussion

The spectrum of severity and its determinants in adult vivax malaria in comparison with falciparum malaria has not been studied adequately. Also, the popular notion of vivax malaria having only ‘benign’ course of infection has been belied [1, 3–7, 11]. Present study aimed to determine the spectrum of severity and its relative contrast between *P. vivax* and *P. falciparum*. This study encompasses the full spectrum (except ‘prostration’) of severity determinants in accordance with the WHO criteria [12]. Although jaundice alone (total bilirubin >3 mg/dL) and thrombocytopaenia are not severity criteria [12, 13], they occur most frequently with all forms of malaria and greatly affect the clinical judgement. Thus, in order to retain the relevance and applicability, liver dysfunction was defined as a rise in total bilirubin ≥2.5 mg/dL with simultaneous three-fold elevation in any serum aminotransferases from their reference upper limits, which is in agreement with contemporary WHO criteria [12].

### Demographic, clinical and laboratory characteristics

Prolonged duration of fever gives an inference of delay in diagnosis/onset of anti-malarial treatment, which could be a potential reason for complications in complicated *P. vivax* cohort. Absence of fever in a significant proportion of complicated *P. falciparum* might have caused delayed presentation, diagnosis and onset of anti-malarial treatment thereby resulting in complicated malaria. Proportion of complicated malaria across all species in the current study varied substantially in comparison to other series (Additional file 3) from diverse geography and endemicity [9]. This variation in severity proportion is apparently also due to lack of uniformity in the definition of severity determinants and in number of determinants included across all studies. Ironically, recent studies [6, 10, 14, 15] have defined severe malaria by severe anaemia (haemoglobin <5 g/dL) and jaundice (total bilirubin >3 mg/dL) with reference to outdated WHO guidelines [16, 17], while revised severity criteria include severe anaemia as haemoglobin <7 g/dL and do not affirm the presence of jaundice alone [12, 13]. This non-uniformity in adoption of severity criteria results in ambiguity, limits the generalizability and use of study outcomes for comparison with other series. Furthermore, variations in spectrum of severity criteria adopted in studies impose difficulties for researchers and policy makers to derive a reliable epidemiological cut-off/index. However, underlying genetic variability determining parasite virulence across diverse geography might also be associated with the variation in the severity proportion across the globe, which requires further exploration.

### Association of complications with mortality by malaria species

Table 1 depicts spectrum of complications and outcome by malaria species. Among severity determinants studied, *P. falciparum* resulted in every complication and, except shock, the rest were clearly associated with mortality. Both hyperparasitaemia and hypoglycaemia did not occur with *P. vivax*, suggesting increased parasite load to be a reason for hypoglycaemia and parasite sequestration, resulting in multiple organ involvement and mortality [12] in *P. falciparum* but not in *P. vivax*. Pulmonary dysfunctions *viz* respiratory distress, PE and ARDS turned out to be the main reasons for mortality in *P. vivax* [6, 15]. Evidence [18–20] is mounting to support that sequestration of *P. vivax* parasites in pulmonary microvasculature results in pulmonary dysfunction; however, further exploration in this direction is certainly warranted. Further, on the spectrum of severity (Additional file 2), *P. vivax* appears to result in relatively lesser amalgamations (up to four) of complications in comparison with both *P. falciparum* (up to seven) and mixed infections (up to six). There is paucity of literature describing a comparable spectrum of complications similar to the current study. Nadkar *et al.* [6] have described up to four organs involved in both *P. vivax* and *P. falciparum*. Sarkar *et al.* [7] have reported up to eight complications and their four combinations with *P. vivax*. Mohapatra *et al.* [10] have described up to four complications in both *P. falciparum* and mixed infections whereas as Tjitra *et al.* [11] have explained only three complications and their combinations across *P. vivax, P. falciparum* and mixed infection. Nonetheless, of the existing studies, it is apparent that the spectrum of complications in malaria is highly variable across the globe. Further multicentric prospective study comprising subjects across the globe, encompassing a uniform and full spectrum of severity determinants, might lead to true realization in this regard.

The present study is in concurrence with those [6, 14, 15, 21, 22] having incremental mortality with increasing organ dysfunctions in *P. falciparum*. Minimal malaria mortality at the study hospital is on par with the best worldwide [14, 21, 23].

### Factors associated with complicated malaria

In contrast with Tjitra *et al.* [11], *P. falciparum* was found to be more likely to cause complicated malaria than *P. vivax*. Rising respiratory rate, falling systolic blood pressure, leucocytosis, and haematuria were independent predictors of severe *P. vivax* malaria. These findings can be used by clinicians as an index to decide intensive care need for the better management and outcome of malaria patients. Occurrence of leucocytosis is inconsistent in all forms of malaria and depends on factors viz. severity of disease, parasitaemia, host immunity and concomitant infections. However, none of the study patients were having bacteraemia or concurrent infections, as this was an exclusion criterion of this study. Thus, either bacteraemia or concurrent infection as a cause of leucocytosis is overruled. Furthermore, low and unstable transmission intensity of malaria in the catchment area of the hospital might be responsible for non-immune status of patients having clinical malaria thus; vigorous inflammatory response might have resulted in baseline leucocytosis. This could be better understood by exploring parasite virulence/immunogenic differences as well as human host specific immune competence between severe and non-severe cohorts. Rising parasite index alone was found to be an independent predictor of severe *P. falciparum*. However, parasite biomass has been advocated to be the superior marker of severity than parasite index due to massive sequestration in *P. falciparum* [24]. Interestingly, despite most of the clinico-laboratory parameters having significant association with disease severity in univariate logistic regression analysis (Table 2), parasite index counterpoised rest all in multivariate logistic regression analysis suggesting hyperparasitaemia to be the determinant of pathophysiology in *P. falciparum*. These differing independent predictors of severity could indicate underlying pathophysiological differences between severe *P. vivax* and *P. falciparum* infections.

### Strengths and weaknesses of the study

The current study has remarkable strengths. This is a unique study describing the full spectrum of severity determinants in accordance with WHO contemporary criteria [12] among *P. vivax* and *P. falciparum* malaria in adult cohort. The study cohort is substantially large comprising >60% *P. vivax*, thereby provides a robust comparison with *P. falciparum* to challenge the ‘benign’ tag with *P. vivax*. Determination of clinico-laboratory parameters as risk factors for severity by multivariate logistic regression analysis provides simple, inexpensive and independent predictors of severity (rising respiratory rate, falling systolic blood pressure, leucocytosis, haematuria and rising parasite index) which could be applied in routine clinical practice. Retrospective design of the study possesses inherent limitations. This study lacks confirmation of the infecting species by polymerase chain reaction. Due to retrospective study design any supportive evidence could not be brought out to the involvement of immune response resulting leucocytosis, thus this must be confirmed and validated through future prospective study design. Furthermore, coagulation parameters and venous bicarbonate levels were not tested in all patients as deemed redundant by the treating physicians. Contribution of pre-existing co-morbidities on disease severity could not be described due to lack of sufficient data; however, none of those expired had any co-morbid etiology. Data on temporal onset of various complications and their progression during illness were not captured. Thus, the pathophysiology of progression of disease from infection to multi-organ involvement to final outcome could not be discerned. Future studies must try to overcome the limitations of this study.

## Conclusions

In adult malaria, the spectrum of severity may be extensively different among various populations with diverse endemicity. Although *P. vivax* does result in ‘severe malaria’ and mortality in considerable proportion, the probability of such is significantly smaller than *P. falciparum*. Furthermore, *P. vivax* results in much lesser amalgamations of multi-organ involvement than *P. falciparum* and mixed infections. Occurrence of PE/ARDS in *P. vivax* infection could lead to mortality, therefore should be diagnosed and treated promptly. Rising respiratory rate, falling systolic blood pressure, leucocytosis and haematuria were the predictors of severity in *P. vivax,* whereas increasing parasite index alone was the predictor of severity in *P. falciparum*. By virtue of higher prevalence of *P. vivax* and substantial proportion of broad spectrum severity as manifested in the current study and other previous series, it is imperative to be vigilant for any probable impositions by *P. vivax* in addition to *P. falciparum*.

## Electronic supplementary material

Additional file 1: **Clinico-demographic and laboratory profile of malaria patients by malaria species.** (XLSX 15 KB)

Additional file 2: **Spectrum of complications with outcome by malaria species.** (XLSX 13 KB)

Additional file 3: **Comparison of present study with other reports describing complicated malaria in adults.** (XLSX 17 KB)
